# Case Report: A case of occupational methyl iodide-induced encephalopathy

**DOI:** 10.3389/fphar.2025.1574692

**Published:** 2025-07-23

**Authors:** Hongyu Liu, Ruikai Shang, Qiaoxin Tian, Yuru Liu, Xiangdong Jian

**Affiliations:** ^1^Department of Poisoning and Occupational Diseases, Emergency Medicine, Qilu Hospital of Shandong University, Cheeloo College of Medicine, Shandong University, Jinan, Shandong, China; ^2^Department of Occupational and Environmental Health, School of Public Health, Cheeloo College of Medicine, Shandong University, Jinan, Shandong, China; ^3^School of Nursing and Rehabilitation, Cheeloo College of Medicine, Shandong University, Jinan, Shandong, China

**Keywords:** iodomethane, poisoning, blurred vision, toxic encephalopathy, occupational exposure

## Abstract

Iodomethane is a commonly used methylation reagent in organic synthesis. However, reports of acute iodomethane poisoning are rare. Herein, we report the successful treatment of a patient with acute iodomethane poisoning. During processing and production, owing to inadequate personal protection, the patient’s eyes were directly exposed to iodomethane gas, leading to blurred vision and magnetic resonance imaging findings consistent with toxic encephalopathy. Following a comprehensive diagnosis and treatment at our hospital, outpatient follow-ups and clinical assessments confirmed the patient’s recovery.

## 1 Introduction

Iodomethane, a colorless volatile liquid with a sweet and unpleasant odor, can serve as a mild warning agent. Acute iodomethane poisoning primarily occurs in the pigment, pharmaceutical, and chemical industries, mostly due to the inhalation of iodomethane vapor during its synthesis and production. The remaining cases are caused by accidental leaks, such as during packaging, transportation, and container ruptures. Although iodomethane is widely used, there are few reports of acute iodomethane poisoning worldwide. The central nervous system is the primary target of acute iodomethane poisoning, with multifocal neurological damage affecting the brain, cerebellum, cranial nerves, and peripheral nerves. Lesions may accumulate in certain cranial nerves. Herein, we report the successful treatment of a patient with acute iodomethane poisoning. During processing and production, owing to the lack of strict personal protection, the patient’s eyes were directly exposed to iodomethane gas, leading to blurred vision and toxic encephalopathy, as observed by magnetic resonance imaging (MRI). Following a comprehensive diagnosis and treatment at our hospital, outpatient follow-ups and clinical assessments confirmed the patient’s recovery.

## 2 Case description

**FIGURE 1:**
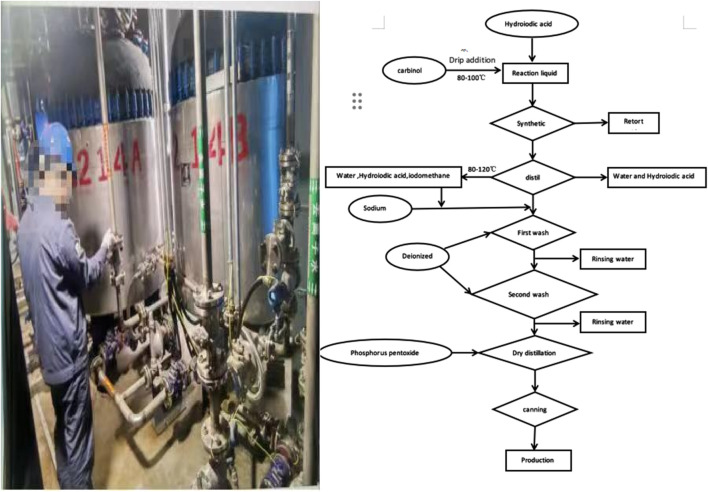
Factory working environment and production flowchart.

The patient was a 38-year-old quality inspector in a chemical plant’s iodomethane synthesis workshop with 9 years of experience and was generally healthy. The workshop’s production process involved the chemical synthesis of iodomethane (Figure 1). The patient reported that on 28 October 2023, when he was inspecting the finished iodomethane product, he did not wear goggles, which directly exposed his eyes to the volatile iodomethane gas. Within a few minutes, he felt nauseous and dizzy, so he immediately left the workplace. Subsequently, he experienced blurred vision and double vision. Before this, the patient had always worn goggles during routine examinations and had never experienced such symptoms. Subsequently, accompanied by colleagues and his wife, the patient was immediately treated at a local hospital, but symptoms such as blurred vision and double vision did not improve. For further treatment, the patient went to our hospital on 31 October 2023. Physical examination findings at the time of admission included: temperature, 36.5°C; pulse, 90 beats per min; respiratory rate, 14 breaths per min; blood pressure, 131/67 mmHg. The patient was conscious, alert, and had clear speech. Both pupils were equal in size, round, with a diameter of 3 mm, and exhibited normal light reflexes. However, he had blurred vision in both eyes, accompanied by diplopia. Bilateral nasolabial folds were symmetrical, and his tongue extended in the midline. Bilateral lung sounds were clear, with no audible dry and wet rales. The heart rate was 90 beats per min, rhythmic, with no pathological murmurs detected in any of the valve areas. The abdomen was flat, with no visible intestinal pattern or gastrointestinal peristalsis wave. There was no tenderness or rebound pain across the entire abdomen. The liver, spleen, and ribs were not palpable, and mobile dullness was absent.

**FIGURE 2:**
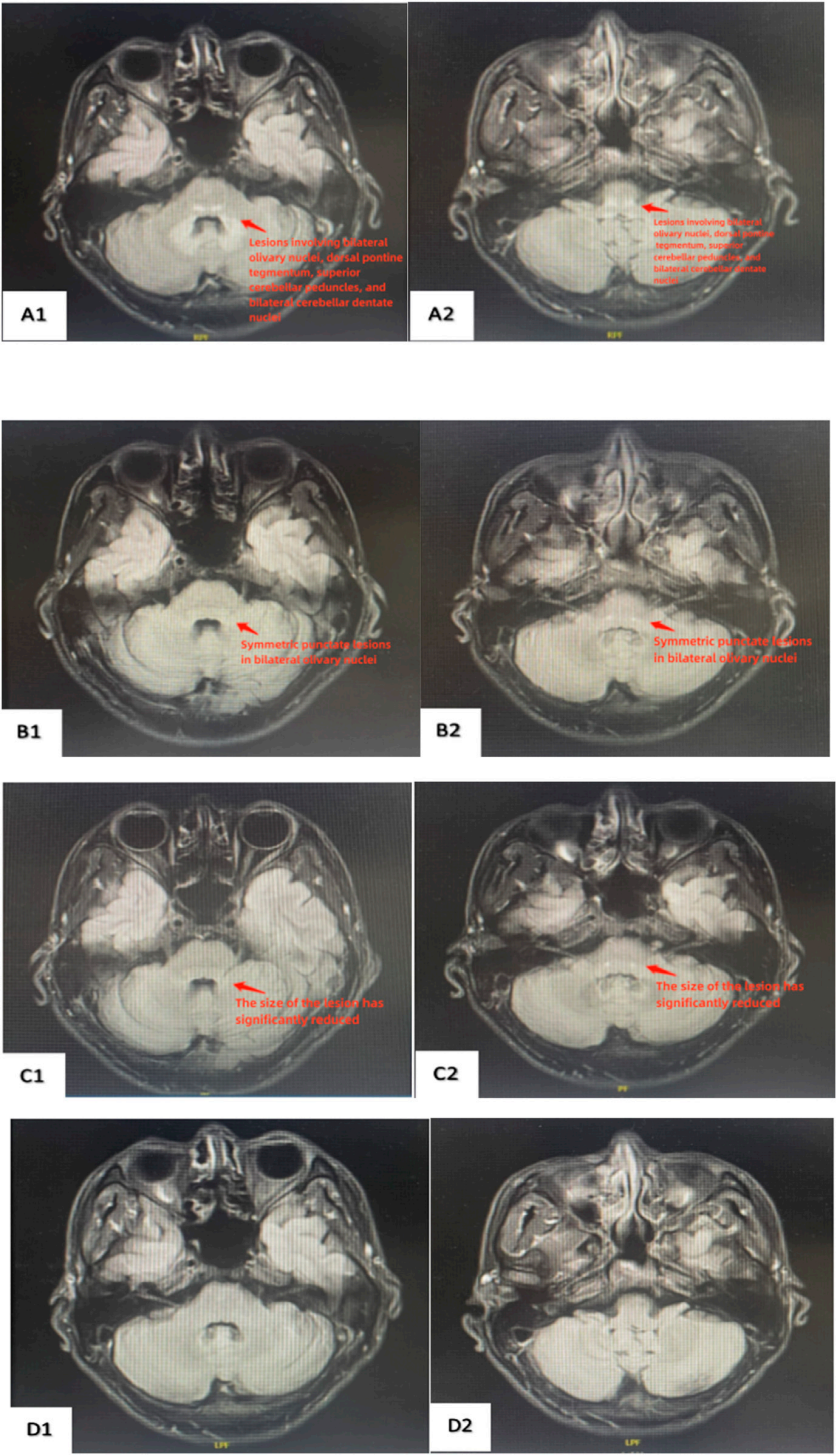
Dynamic changes in brain MRI before and after treatment. **(A1,A2)** (2023.11.4): Symmetrical abnormal signals in the bilateral olivary nuclei, tegmental pontine region, superior cerebellar peduncles, and bilateral dentate nuclei of the cerebellum. **(B1,B2)** (2023.11.13): Symmetrical abnormal signals in the bilateral olivary nuclei. **(C1,C2)** (2023.11.20): The abnormal symmetrical signals in the bilateral olivary nuclei have significantly decreased in size compared to previous scans, with some areas no longer clearly visible. **(D1,D2)** (2024.3.12): Abnormal symmetry signal of bilateral olivary nuclei have disappeared.

**FIGURE 3:**
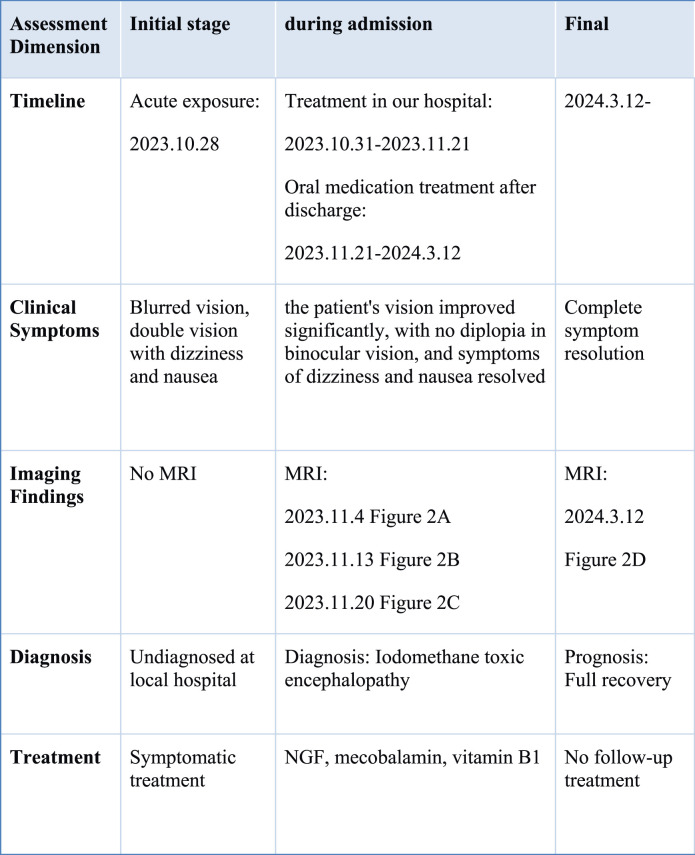
Timeline with a table.

Physiological reflexes were present, and no pathological reflexes were elicited. Preliminary diagnosis: iodomethane poisoning. Routine blood tests, liver and kidney function tests, coagulation profile, and other laboratory indicators were normal initially. Head and neck MRI revealed symmetrical patchy long T1 and long T2 signals in the bilateral olivary nuclei-dorsal pontine tegmentum-superior cerebellar peduncles and bilateral cerebellar dentate nuclei, with hyperintensity on T2 FLAIR and isointensity on DWI, consistent with the MRI findings of toxic encephalopathy (Figure 2A). Upon admission, the patient was treated with nerve growth factor, mecobalamin, and vitamin B1. After 2 weeks of pharmacotherapy, the patient’s vision improved significantly, with no diplopia in binocular vision, and symptoms of dizziness and nausea resolved. On 13 November 2023, follow-up head and neck MRI demonstrated symmetrical punctate long T1 and long T2 signals in the bilateral olivary nuclei, with hyperintensity on T2 FLAIR (Figure 2B). The patient continued hospitalization for pharmacotherapy. On 20 November 2023, the laboratory tests continued to be normal. Head and neck MRI revealed symmetrical punctate long T1 and long T2 signals in bilateral olivary nuclei with hyperintensity on T2 FLAIR, demonstrating a significant reduction in lesion size compared to the previous MRI on 13 November 2023 (Figure 2C). After hospitalization, the patient’s symptoms improved significantly, and head and neck MRI showed a marked reduction in lesion size. The patient was discharged on 21 November 2023, and was prescribed oral mecobalamin for follow-up treatment with regular outpatient visits. On March 12 of the following year, the patient attended an outpatient follow-up. The laboratory tests continued to be normal. Head and neck MRI showed no abnormal signals in the brain parenchyma, normal morphology and signals in the ventricular cistern fissure system, midline structures in the middle, and complete resolution of the lesion (Figure 2D). The patient’s vision fully recovered to normal. The patient's initial stage, during admission and final outcome have been summarized in a timeline table (Figure 3).

This study was approved by the Ethics Committee of Qilu Hospital of Shandong University, and informed consent was obtained from all patients.

## 3 Discussion

Iodomethane, with the molecular formula CH_3_I, is a colorless liquid under normal temperature and pressure. It turns yellow, red, or brown when exposed to light and moisture, and has a strong ether-like odor. The boiling point is 42.4°C. It is soluble in ethanol, ether, acetone, and benzene, and slightly soluble in water. It is commonly used as a raw material for the production of drugs such as iodomethyl methionine (vitamin U), analgesics, and antidotes, as well as raw materials for fire extinguishers and other organic synthetic compounds. It can also be used as a methylation reagent in the synthesis of pyrazoles and in microscopic examinations.

Acute iodomethane poisoning primarily occurs in the pigment, pharmaceutical, and chemical industries, mostly due to the inhalation of iodomethane vapor during its synthesis and production. In cases of accidental exposure, acute iodomethane poisoning primarily targets the central nervous system (CNS), characterized by multifocal neurological damage involving the brain, cerebellum, cranial nerves, and peripheral somatic nerves. The affected sites generally correlate with clinical manifestations (Hermouet et al., 1996). In addition to CNS injury, multiple organ damage can occur, including the respiratory system, heart and kidneys. Upon massive inhalation of iodomethane vapor, patients may develop edema and necrosis of tracheobronchial mucosa, as well as burns to respiratory mucosa, leading to ventilatory dysfunction and dyspnea. Most poisoned patients show no significant electrocardiogram (ECG) changes. Patients with severe acute iodomethane poisoning may exhibit decreased blood carbon dioxide combining power, increased blood urea nitrogen (BUN), reduced creatinine clearance rate, oliguria, and decreased serum potassium. Individual cases may show positive urine protein and erythrocyturia in urinalysis (Appel et al., 1975).

Occupational acute iodomethane poisoning primarily occurs during the synthesis and production of iodomethane in the pharmaceutical and chemical industries. Although iodomethane is synthesized and used in various regions of our country, reports of acute iodomethane poisoning remain scarce (Schwartz et al., 2005; Nair and Chatterjee, 2010). Acute iodomethane poisoning mainly affects the central nervous system and is characterized by multifocal neurological damage involving the brain, cerebellum, cranial nerves, and peripheral nerves. Lesions accumulate in certain cranial nerves, primarily presenting as blurred vision, diplopia, strabismus, retrobulbar optic neuritis, dysarthria, and hearing loss, which may be associated with damage to the third, fourth, sixth, and eighth cranial nerves (Hermouet et al., 1996). Patients initially develop central nervous system symptoms after inhaling high concentrations of iodomethane vapor over a short period. In severe cases, brain MRI scans reveal white matter and basal ganglia lesions along with abnormal signals in the middle cerebellar peduncle and splenium of the corpus callosum. GSH depletion is the primary cause of both acute and chronic neurotoxicity induced by halogenated methanes (Hermouet et al., 1996). *Ex vivo* studies have confirmed that GSH depletion is the sole mechanism underlying iodomethane-induced neurotoxicity (Chamberlain et al., 1999). Culture experiments on nerve cells, including glial cells and the neuron-glial cell system, have demonstrated the GSH-depleting effect of iodomethane (Bonnefoi et al., 1991). Studies have also shown that iodomethane toxicity increases as intracellular GSH levels decline. In contrast, pre-treatment with GSH before iodomethane provides neuroprotection (Bonnefoi, 1992; Davenport et al., 1992). Animal experiments have shown that the toxic effect of iodomethane is induced by inhibiting brain glutathione enzymes, leading to glutathione depletion. When mouse cerebral cortical cell cultures were exposed to iodomethane for 5 min, cellular damage was associated with the loss of mitochondrial GSH (Bonnefoi et al., 1991). Glutathione depletion weakens the cell’s ability to scavenge free radicals, placing cells in a unique environment prone to oxidative stress damage. Free radicals can also cause disorders in cellular energy metabolism, leading to cell injury. Pretreatment with antioxidants significantly alleviates cellular injury (Hermouet et al., 1996).

Iodomethane poisoning is characterized by the gradual onset followed by the sudden exacerbation of central nervous system damage after excessive exposure to iodomethane vapor, with symptoms appearing within 2–72 h. In this case, after exposure to iodomethane vapor, the patient developed symptoms such as blurred vision, and multiple head and neck MRI scans during hospitalization revealed findings consistent with toxic encephalopathy. The patient’s nervous system injury was likely caused by iodomethane poisoning. Mouse Nerve Growth Factor (mNGF), vitamin B1 and mecobalamin support nerve regeneration and repair. Nerve growth factor, the first discovered neurotrophic factor, plays a pivotal role in maintaining neuronal survival, growth, differentiation, and facilitating repair and regeneration after nerve injury (Petruska and Mendell, 2004). Experiments have shown that mNGF can improve the neurogenesis and inhibit neural apoptosis in the hippocampus following HIBD in a neonatal rat model (Yin et al., 2013). The neurotropic B vitamins thiamine (B1) and cobalamin (B12) are key players, which maintain the neuronal viability in different ways. They constantly protect nerves against damaging environmental influences. Vitamin B1 acts as a site-directed antioxidant and vitamin B12 maintains myelin sheaths. When nerve damage occurs, the presence of vitamins B1 and B12 paves the way out to the following important regeneration by supporting the development of new cell structures. Furthermore, vitamin B1 facilitates the usage of carbohydrates for energy production, whereas vitamin B12 promotes nerve cell survival and remyelination (Baltrusch, 2021).

Based on the worksite data provided by the patient and the investigation of their occupational exposure history, it was determined that the patient was exposed to excessive iodomethane gas at work. This case highlights the need for enterprises involved in iodomethane production to implement occupational health training, enhance employee awareness of protective measures, and conduct regular health examinations for workers with long-term iodomethane exposure. These measures can help identify potential risks early and prevent poisoning incidents.

## Data Availability

The original contributions presented in the study are included in the article/supplementary material, further inquiries can be directed to the corresponding author.
